# Results of omalizumab treatment in chronic eosinophilic pneumonia: Real-life data

**DOI:** 10.5578/tt.202401795

**Published:** 2024-03-26

**Authors:** Buket BAŞA AKDOĞAN, Kurtuluş AKSU, İlkay KOCA KALKAN, Gözde KÖYCÜ BUHARİ, Özlem ÖZDEDEOĞLU, Hale ATEŞ, Ferda ÖNER ERKEKOL

**Affiliations:** 1 Clinic of Immunology and Allergy, Health Sciences University Atatürk Chest Diseases and Thoracic Surgery Training and Research Hospital, Ankara, Türkiye

## Abstract

**ABSTRACT**

**
Results of omalizumab treatment in chronic eosinophilic pneumonia: Real-life data
**

**Introduction:** *Recurrences occur when corticosteroid therapy is discontinued or reduced during the treatment of chronic eosinophilic pneumonia (CEP). The probability of recurrence is once in 50% of patients and twice or more in 25%. In such instances, new treatment options are deemed necessary. This study aims to assess the efficacy of omalizumab treatment as a steroid-sparing drug in patients with CEP.
*

**Materials and Methods:** *The clinical features of patients treated with omali- zumab for recurrent CEP were evaluated retrospectively before and after treatment. All data from patients and diagnoses were reviewed. The effects of treatment on recurrence rate, oral corticosteroid (OCS) use and lung func- tions, peripheral eosinophil values, and symptom scores were evaluated. Radiological regression was also evaluated.
*

**Results:** *In the final analysis, we included ten patients with a median follow-up of 22 months after initiation of omalizumab. During this follow-up period, the results were associated with a significant reduction in the number of asthma attacks per year, the number of CEP relapses, the rate of hospitalization, the amount of corticosteroids consumed daily, and the total corticosteroid dose. In addition, improvement was observed in the symptom scores and lung func- tions of the patients. Systemic steroids were completely discontinued in two patients receiving omalizumab treatment. In other patients, the mean steroid dose was reduced by 77.2 percent in the first year of omalizumab treatment and 82 percent in the second year, respectively. Nevertheless, there was no elevation in peripheral eosinophil count, and radiological regression was observed.
*

**Conclusion:** *Omalizumab can be an effective treatment for CEP and can be used as a steroid-sparing agent.
*

**Key words:** *CEP; omalizumab; mepolizumab; steroid-sparing agent
*

**ÖZ**

**
Kronik eozinofilik pnömonide omalizumab tedavisinin sonuçları: Gerçek yaşam verileri
**

**Giriş:** *Kronik eozinofilik pnömoni (KEP) tedavisinde, kortikosteroid tedavisi kesildiğinde veya azaltıldığında nüksler meydana gelir. Nüks olasılığı hastaların %50'sinde bir kez, %25'inde ise iki kez veya daha fazladır. Bu durumda yeni tedavi seçeneklerine ihtiyaç duyulmaktadır. Bu çalışmanın amacı, steroid koruyucu bir ilaç olarak omalizumab tedavisinin KEP'li hastalardaki etkililiğini değerlen- dirmektir.
*

**Materyal ve Metod:** *Rekürren KEP nedeniyle omalizumab ile tedavi edilen hastaların klinik özellikleri tedavi öncesi ve sonrası retros- pektif olarak değerlendirildi. Hastaların tüm verileri ve tanıları gözden geçirildi. Tedavinin nüks oranı, oral kortikosteroid (OKS) kulla- nımı ve akciğer fonksiyonları, periferik eozinofil değerleri ve semptom skorları üzerindeki etkileri değerlendirildi. Radyolojik regresyon değerlendirildi.
*

**Bulgular:** *Son değerlendirmeye, omalizumab başlandıktan sonra medyan takip süresi 22 ay olan 10 hasta dahil edildi. Bu takip süre- si boyunca sonuçlar, yıllık astım atağı sayısında, KEP nüks sayısında, hastaneye yatış oranında, günlük tüketilen kortikosteroid mikta- rında ve toplam kortikosteroid dozunda anlamlı bir azalma ile ilişkilendirildi. Ayrıca hastaların semptom skorlarında ve akciğer fonk- siyonlarında iyileşme gözlendi. Omalizumab tedavisi alan iki hastada sistemik steroidler tamamen kesilmiş, diğer hastalarda ise orta- lama steroid dozu omalizumab tedavisinin birinci yılında %77,2, ikinci yılında ise %82 oranında azaltılmış, buna rağmen periferik eozinofil sayısında artış olmamış ve radyolojik gerileme saptanmıştır.
*

**Sonuç:** *Omalizumab, KEP için etkili bir tedavi olabilir ve steroid koruyucu bir ajan olarak kullanılabilir.
*

**Anahtar kelimeler:** *KEP; omalizumab; mepolizumab; steroid koruyucu ajan
*

## INTRODUCTION

Eosinophilic pneumonia (EP) is a rare condition among diffuse parenchymal lung diseases. It is characterized by marked eosinophil accumulation in the alveolar space and interstitium. Significant eosinophil accumulation is observed in lung tissues and/or bronchoalveolar lavage (BAL) fluid. Although chronic eosinophilic pneumonia (CEP) responds well to corticosteroids, relapses are common. Therefore, corticosteroids may not be discontinued in treatment (1). The annual incidence of CEP is estimated to be

0.23 cases/100.000 population, but the exact incidence of CEP in the general population has not been determined (2). The diagnosis is made according to established diagnostic criteria.

Omalizumab reduces the level of free IgE in the circulatory system, down-regulates high-affinity IgE receptors (FcεRI) in inflammatory cells, and has been used in the treatment of many diseases, including asthma, chronic urticaria, eosinophilic gastrointestinal disorders (EGIDs), allergic rhinitis and allergic bronchopulmonary aspergillosis (ABPA) (3-11). They are very effective in the treatment of patients with allergic asthma. In a study evaluating its efficacy in adolescents with uncontrolled moderate to severe allergic asthma, Omalizumab was associated with improved lung function and a decrease in the number of circulating eosinophils (12-15).

Since relapses may occur when systemic corticosteroid therapy is discontinued or reduced in the treatment of CEP, the use of steroid-reducing agents was

investigated in this study. There are cases in the literature where CEP was successfully treated with anti-IgE therapy (13). There have also been successful treatment response outcomes observed with other biological agents including mepolizumab and benralizumab; however, these experiences are limited (1,11,13-18).

In our study, we assessed the outcomes of omalizumab treatment, a long-standing steroid-sparing medication that we believe to be effective, in patients with CEP.

## MATERIALS and METHODS

**Study Design**

The records of patients diagnosed with CEP who received omalizumab treatment at the adult allergy and immunology clinic of our hospital between June 2011 and June 2017 were retrospectively reviewed by the designated investigators. Our hospital is a tertiary chest diseases and thoracic surgery facility and serves as a reference center in its field. Our study was approved by the Medical Specialization Education Board and the Local Ethics Committee (Approval number: 08-2018/1744). Informed consent forms were obtained from all patients before treatment. In addition, approval was obtained from the Ministry of Health Turkish Medicines and Medical Devices Agency for the off-label use of the drug.

## Cases

The demographic and clinical characteristics of the patients were extracted from their medical records.

Başa Akdoğan B, Aksu K, Koca Kalkan İ, Köycü Buhari G, Özdedeoğlu Ö, Ateş H, et al.

Patients who presented with relevant clinical symptoms and fulfilled all of the following diagnostic criteria were considered to have CEP. All patients also had a diagnosis of nonatopic asthma.

1. Pulmonary alveolar consolidation, especially peripherally located, with air bronchograms and/or ground-glass opacities on radiological imaging.
2. 25-40% total eosinophils in differential cell count in bronchoalveolar lavage (BAL) (or 1000/mm^3^ eosinophils in peripheral blood).
3. Presence of respiratory symptoms for at least two to four weeks.
4. Exclusion of other known causes of eosinophilic lung disease (1,2,19-21).

Systemic corticosteroid therapy was initiated upon diagnosis in patients with CEP. They were using asthma treatment, per GINA guidelines. Relapses and asthma attacks of the patients were evaluated. An exacerbation of asthma symptoms necessitating three days or less of systemic steroid therapy, along with an escalation in bronchodilator therapy dosage, was classified as an asthma attack. In general, the escalation of pulmonary symptoms, decline in respiratory function, rise in peripheral eosinophilia count, and concurrent radiological findings such as consolidation or ground glass opacities-frequently observed upon tapering steroid doses to 7.5 mg or below-were assessed as indicative of CEP recurrence.

Patients who experienced two or more recurrences of CEP during follow-up, or one recurrence of CEP along with at least two asthma attacks within the last year, and who demonstrated uncontrolled asthma or required maintenance therapy with high-dose oral corticosteroids (OCS) to achieve control, leading to frequent unscheduled physician visits, emergency visits, and/or serious complications associated with systemic steroid use, were identified as eligible candidates for steroid-sparing omalizumab treatment.

Omalizumab dose (Xolair, Novartis, Sweden) and total immunoglobulin (Ig) E levels and weights of the patients were calculated from the table. The dosages administered were as follows: 600 mg every two weeks for two patients, 450 mg every four weeks for one patient, 300 mg every four weeks for six patients, and 150 mg every four weeks for one patient. Routine clinical and functional follow-ups were conducted at our clinic.

## Measurements

All perennial allergens (house dust, mold, cockroach, cat) and pollen were included in the DPT used in the skin prick test (ALK, Abello, Spain) to assess patients’ sensitivity to inhaled allergens. Total IgE and Specific IgE measurement results (Synlab, PhadiaUniCAP, Uppsala, Sweden) were used. PA Chest X-ray and high-resolution computed tomography (HRCT) (GE Healthcare, USA) were planned for patients with compatible clinical findings. A hemogram analysis comprising 22 parameters was conducted (Mindray BC 5380, CHINA). Patients exhibiting clinically appropriate radiological findings underwent bronchoalveolar lavage (BAL) and flexible fiberoptic bronchoscopy procedures (Olympus, Japan). Differential cytological analyses were performed on BAL fluid samples. All patients underwent fecal parasite examination to investigate other causes of peripheral eosinophilia. The patients had no history of drug use known to induce eosinophilia. Collagen markers (ANA, ANCA, etc.) were examined to identify collagen tissue diseases. To exclude organ involvement, patients underwent cardiological and neurological examinations. Abdominal ultrasonography (USG) and echocardiography (ECHO) evaluations were performed. None of the patients were diagnosed with EGPA, ABPA, or HES. Patients were referred to necessary units for consultation. Asthma control tests (ACT) and pulmonary function tests (PFT) (ZAN 100, Germany), including measurements of forced expiratory volume

per second (FEV1), forced vital capacity (FVC), and the FEV1/FVC ratio, were conducted for all patients who regularly attended our clinic for omalizumab

injections.

Patients were receiving a daily dose of steroids and inhaled corticosteroids (ICS). The daily steroid dose before initiating omalizumab, specifically before the most recent relapse, was documented. To determine the total oral corticosteroid (OCS) dose, the amount of OCS administered to patients over the past year, including during exacerbations and relapses, was calculated based on daily doses. Asthma exacerbations, relapses, and hospitalizations were documented for one year before and one year after commencing omalizumab treatment.

In the evaluation of the data, it was found that all patients (n= 10) used omalizumab for the first 16 weeks. Nine patients continued omalizumab treatment for at least one year, while one patient was

lost to follow-up at six months due to personal reasons. Data from five patients who received omalizumab for two years and four out of the ten patients who received omalizumab for three years were recorded. Patients whose data could not be collected and those who did not fully meet the diagnostic criteria were excluded from the study.

## Outcomes

The efficacy of omalizumab treatment was assessed based on symptom control of the patients, daily dose of OCS, total annual dose of OCS, improvements in PFTs and reduction in asthma exacerbations, number of CEP relapses, and hospitalizations.

Patients who maintained symptom control without experiencing exacerbations, relapses, or hospitalizations, and who demonstrated a reduction in daily OCS doses or clear improvement in PFTs, were considered to be fully responsive to treatment. If at least one of the above parameters could not be achieved, the patient was considered to have a partial response. If none of the parameters could be achieved, the patient was considered unresponsive to treatment. Response to treatment was evaluated by all authors or physicians involved in the clinical follow-up of the patients.

## Statistical Analysis

The statistical analysis was performed using SPSS version 20.0 (SPSS Inc., Chicago, IL, USA). Numerical values with normal distribution were expressed as means ± SD and non-normal variables with distribution were represented as median values (min- max). Categorical variables were represented as n (%). Comparisons were performed using the Wilcoxon signed-rank test before treatment (pre-omalizumab) and after treatment with omalizumab. The difference in the average total amount of OCS administered per patient for 12 months from the onset of omalizumab treatment (post-omalizumab or pre-omalizumab) was also tested using Wilcoxon. All p values were bi-directional, and p< 0.05 was considered the limit of significance.

## RESULTS

**Demographic and Disease Characteristics**

The data from 10 patients (five women, five men) with a mean age of 38.5 (23-44 years) were evaluated. The

median body mass index of the patients was 25.1 kg/ mZ (18.7-33.1). All patients had a diagnosis of asthma. The median duration of asthma was 76.6 (4-144) months. All subjects had negative SPT and specific IgEs. Case 5 had hypertension. Seventy percent of the patients had comorbidities (such as OSA, CRSwNP, NSAID allergy, and GER). In addition, the rates of nasal polyposis and nonsteroidal anti-inflammatory drug (NSAID) hypersensitivity were 40 percent and 30 percent, respectively. Effective beta-agonists were administered. Moreover, 70% of the patients were receiving treatment with montelukast. All patients had a history of frequent systemic corticosteroid use. Corticosteroid side effects were observed in 90 percent of the patients (cataracts, cushingoid facial appearance, pityriasis versicolor, osteoporosis). None of the patients were active smokers. Three patients were former smokers (Table 1).

## Assessment of Subjects

The initial symptoms of the patients were consistent with CEP. All patients presented with peripheral eosinophilia exceeding 1000/mm^3^, with a median count of 2550 eosinophils/mm^3^ in peripheral blood (1200-3900) (Table 2). In all cases, radiological evaluations (PA or HRCT) revealed the presence of consolidation areas and ground glass opacities (Figure 1). FOB was performed in nine subjects. The procedure could not be performed on patient #9 due to their overall medical condition. BAL eosinophil levels were 32 (3-65) µL. Patients without significant eosinophilia in BAL exhibited high values of peripheral eosinophilia. The diagnosis of CEP was confirmed. Fecal parasites were evaluated and found negative in all patients. The mean total IgE level was

355 ± 417 IU/mL. Nine subjects had negative collagen markers, and patient #8 had a positive p-ANCA of 1/20. There was no organ involvement in any of the patients. Oral methylprednisolone (0.5-1 mg/kg/day) was administered to each individual at the time of diagnosis. The mean annual corticosteroid dose was 4865 ± 1312 mg, and the daily corticosteroid dose was 7.4 ± 4.9 mg. The mean clinical recurrence rate was 3.2 ± 1.2 and the mean hospitalization rate was 1.1/year when the corticosteroid dose was reduced during follow-up. Subjects were treated with omalizumab to capitalize on its steroid-sparing effect and maintain control of asthma symptoms. The mean onset time of omalizumab was 29.2 ± 10.2 months.

**Table:** 

**Table 1.** Demographic and clinical characteristics of the patients																
**PatIent/ sex/Age**	**Smoking** **+/-**	**Comorbidity** **+/-**	**Per eos**	**BAL** **eos**	**Total IgE kU/L**	**Radiology finding (PA-HRCT)**	**Atopy/ Asthma**	**Omalizumab dose (mg)/** **treatment**	**ACT** **b/a**	**Exac./ year (b/a)**	**Rec/ year b/a**	**Hosp./ Year (b/a)**	**OCS** **dose mg/day (b/a)**	**Total OCS dosage mg/year (b/a)**	**FEV1%/** **mL (b/a)**	**FVC%** **(b/a)**
1. F/27	-	+	1200	34	38	-/2	-/+	150 mg/4w	16/21	3/1	3/0	1 /0	4/2	3880/564	74/88	92/116
2. F/27	-	-	1300	4	153	+/2	-/+	300 mg/4w	9/22	6/0	3/0	2/0	16/4	6820/1560	64/90	77/96
3. F/44	+	+	1200	40	251	-/2	-/+	300 mg/4w	13/25	2/0	2/0	1/0	8/0	3338/0	80/105	86/99
4. M/23	-	+	3100	50	181	-/2	-/+	300 mg/4w	11/25	2/0	1/0	1/0	2/0	4300/120	59/80	74/91
5. F/43	-	+	3000	5	178	+/1	-/+	300 mg/4w	12/22	3/1	1/0	1/0	4/1	3510/540	88/90	92/95
6. M/41	-	+	3100	5	107	-/1	-/+	300 mg/4w	18/22	4/0	3/1	1/1	8/4	6300/2320	71/79	69/84
7. F/38	-	-	2100	65	1120	-/1	-/+	600 mg/2w	12/24	2/2	2/0	1/0	8/1	6744/1230	58/97	63/99
8. M/39	-	+	3500	32	1086	+/2	-/+	600 mg/2w	15/25	3/0	2/0	1/0	16/4	4618/1542	116/119	111/109
9. M/40	+	-	3900	-	117	+/1	-/+	300 mg/4w	18/12	3/1	2/1	1/1	4/4	5020/2190	77/59	87/72
10. M/30	+	+	1400	3	225	+/1	-/+	450 mg/4w	18	3/-	1/-	1/-	4/-	4120/-	78/-	97/-
F: Female, M: Male, with/without smoking history, Comorbidities: Chronic sinusitis, chronic sinusitis and nasal polyp, drug allergy, food allergy, venom allergy, sleep apnea syndrome, gastroesophageal reflux, patient number 5 had hypertension. Per eos: Peripheral eosinophilia, BAL eos: Percentage of bronchoalveolar lavage eosinophilia Radiology finding (PA chest X-ray presence/absence of significant infiltration in PA chest X-ray significant infiltrating area. HRCT: High-resolution computed tomography unilateral= 1 /bilateral= 2/ no finding= 0. Oma: Omalizumab, omalizumab dose, ACT: Asthma con- trol test. ACT is a questionnaire consisting of five questions. Patients rate each question between one and five points. The total score of the five questions forms the test result. If the total score is 25, asthma is considered under full control, 24-20 demonstrates partial control, and ≤19 means asthma is not under control. Exac: Asthma exacerbation, asthma attack requiring oral steroids for less than three days), Rec: Recurrence (as explained in the text) Hosp: Hospitalization, b: Before omalizumab, a: First year after omalizumab, y: Year, m: Month, w: Week, FEV1: Forced expiratory volume, FVC: Forced vital capacity. **Patient 10 was lost to follow-up six months after starting Omalizumab.																

**Table:** 

**Table 2.** Patients’ characteristics	
Gender M/F	5/5
Age, years (median, range)	38.5 (23-44)
Blood eosinophils at diagnosis, n/μL (median, range)	2550 (1200-3900)
BAL eosinophil percentage at diagnosis (median, range)	32 (3-65)ᵃ
FVC at baseline, % predicted (median, range)	86.5 (63-111)
FEV1 at baseline, % predicted (median, range)	75.5 (58-116)
FVC: Forced vital capacity, FEV1: Forced expiratory volume in 1 sec. a One missing data

**Figure 1.** Radiological findings.

Representative example of baseline HRCT and PA chest radiography lesions compared to control HRCT after one year of omalizumab treatment showing the disappearance of ground glass opaci- ties and consolidations A) Before treatment B) After omalizumab treatment.

## Assessment of omalizumab response

At the time of data collection for the study, nine patients had completed the first year of treatment, five patients had completed the second year, and four patients had completed the third year. Baseline values for ACT, FEV1%, FEF25–75%, and peripheral eosinophil counts before the start of omalizumab treatment, as well as at the sixteenth week, first year, second year, and third year of omalizumab treatment are provided in Table 3**.**

During omalizumab treatment follow-up, despite the reduction in systemic corticosteroid dosage, mean ACT scores significantly increased at the sixteenth week and during the first and second years. Subsequently, FEV1% values improved in a statistically significant manner during the first year of omalizumab treatment. Moreover, there was no significant change in eosinophil counts despite the decrease in systemic corticosteroid dose (as demonstrated in Table 3 and Figure 2B). Patients also exhibited radiological regression. Data from a patient is provided as an example.

**Table:** 

Table 3. Clinical and functional characteristics of cases recorded before and after the start of omalizumab								
	**ACT score**	**p**	**FEV1%ƅ**	**p**	**FVC%**	**p**	**FEF%25-75 C**	**p**
Before treatment (n= 10)	14 (9-16)		75.5 (58-116)		86.5 (63-111)		50.5 (63-111)	
16th week (n=10)	23.5 (19-25)	**0.005**	95.5 (76-128)	**0.012**	96.5(86-125)	**0.01**	92 (47-152)	**0.009**
1st year (n= 9)	22 (12-25)	**0.02**	90 (59-119)	**0.038**	96 (72-116)	0.64	73 (34-136)	0.192
2nd year (n= 5)	25 (12-25)	**0.04**	85 (65-108)	0.13	98 (67-113)	0.2	65 (48-93)	0.080
3rd year (n= 4)	25 (22-25)	0.068	84.5 (76-85)	0.068	94.5 (91-101)	0.19	52.5 (39-60)	0.465
Date is presented as median (min–max) unless stated otherwise. a ACT: Asthma control test. A total score of 25 is considered complete control, 24-20 partial control, and <19 not under control. b Forced expiratory volume in FEV1 is the amount of air expelled from the lungs in liters in the 1st second of the forced expiratory maneuver c FEF25-75%maximal mid expiratory flow rate MMFR p< 0.05 was considered statistically significant. d Forced vital capacity (FVC): It is the volume of gas that can be removed from the lungs with a forced, rapid and deep expiration following a deep and difficult inspiration using effort.

**Figure 2A.** Follow-up graphics of patients.

**A.** Eosinophil levels, **B.** Average daily steroid use of cases, **C.** CEP recurrence, hospitalization, and frequency of asthma attacks (the term “before treatment” reflects the treatment and follow-up values of patients who received steroid therapy before starting omali- zumab treatment).

**2B.** Follow-up graphics of patients.

**A.** Pulmonary function tests FEV1 percent values are given, **B.** Asthma control test (ACT) results of the patients; ACT is a questionnaire that consists of five questions. Patients rate each question between one and five points. The total score of the five questions forms the test result. If the total score is 25, asthma is considered under full control, 24-20 demonstrates partial control, and ≤19 means asthma is not under control.

Following the administration of omalizumab treatment, there was a significant decrease observed in the rates of asthma exacerbations, recurrence of the disease, and hospitalizations. While the mean recurrence rate had been identified to be two per year before omalizumab treatment, it was observed to be 0.22 per year in the first year of omalizumab treatment (n= 9, p= 0.007). Similarly, the hospitalization rate decreased from 1.1 per year to

0.22 per year (n= 9, p= 0.01), and the frequency of exacerbations decreased from 3.1 per year to 0.55 per year (n= 9, p= 0.01) (as highlighted in Figure 2A).

## Systemic corticosteroid use during omalizumab treatment

The daily OCS dose of the patients decreased after the initiation of omalizumab treatment. Treatment

with systemic steroids was completely discontinued at the sixteenth week in two patients. The mean total dose of corticosteroids in other patients decreased by 77 percent in the first year of treatment and by 82 percent in the second year of treatment (as shown in Table 4).

## DISCUSSION

In our study, we present valuable data on the efficacy of omalizumab, a medication that has been utilized extensively over time. However, we acknowledge that the data may be limited specifically in cases of chronic eosinophilic pneumonia (CEP). We believe that omalizumab, as a steroid-sparing medication, demonstrates effectiveness in the treatment of CEP. During follow-up, it was observed that omalizumab reduced the frequency of relapses, minimized

**Table:** 

**Table 4.** Systemic corticosteroid use and eosinophil levels before and after omalizumab treatment						
	**Daily steroid dose (mg/day)**	**p**	**Total steroid dose (mg/year)**	**p**	**Eosinophil level**	**p**
Before treatment (n= 10)	6 (2-16)		4459 (3328-6820)		650 (101-2000)	
16th week (n= 10)	3 (0-4)	**0.007**	-		-	
1st year (n= 9)	2 (0-4)	**0.012**	1230 (0-2320)	**0.008**	550 (300-1900)	0.59
2nd year (n= 5)	1 (0-4)	0.068	770 (200-1920)	0.04	648 (300-1040)	0.22
3rd year (n= 4)	1 (0-4)	0.1	720 (0-3364)	0.068	709 (426-1060)	0.71
Before treatment; period before omalizumab treatment. Date is presented median (min-mx) unless stated otherwise. p< 0.05 was considered statis- tically significant.

hospitalizations, decreased the number of asthma exacerbations, and maintained stable eosinophil levels despite the reduction or cessation of steroid therapy. Additionally, patients experienced improvements in symptom control and pulmonary function.

Carrington et al. were the first to describe CEP (22). The absence of specific symptoms unique to the disease often leads to diagnostic challenges (20). Asthma accompanies the disease in more than 50 percent of cases (19-21). Eosinophilic lung diseases should also be considered when systemic symptoms such as unexplained fever, cough, weight loss, and back pain accompany asthma symptoms, when the number of exacerbations is high during asthma treatment, and when the cause of exacerbation is unclear (19-21). Defined diagnostic criteria must be met for diagnosis of CEP. However, it is important to note that there are variable diagnostic criteria defined in the literature. There are definitions of peripheral eosinophilia ranging from #500/mm^3^ eosinophils in peripheral blood to >10% and BAL eosinophil percentages ranging from 5% to 40% (19-21). Since CEP is also a diagnosis of exclusion, there is no standardization in the diagnosis and therefore in the study data (23). We used the diagnostic criteria described in detail in the Methods section. The incidence of the mirror image description of pulmonary edema in CEP patients is around 25%, and the rate of peripheral consolidation is 50%. Unilateral infiltration can be detected in up to 20% of cases, and indeed, we had patients exhibiting unilateral infiltration in our study cohort. BAL eosinophilia was found to be quite high, reaching levels such as 65%. Radiologically, peripheral consolidation areas and ground glass attenuation areas were observed in the patients, which were

found to be consistent with chronic eosinophilic pneumonia (CEP). As an example, CT images of one of our patients before and after omalizumab treatment are presented in Figure 1 (1,22). In the literature, it is reported that bronchoalveolar lavage (BAL) eosinophilia can surpass transbronchial biopsy (TBB) as a diagnostic tool for CEP. TBB was not obtained in our patients. In the literature, it is thought that BAL eosinophilia may be more diagnostically significant than TBB results for the diagnosis of CEP. In the study by Matsuse et al., tissue eosinophilia was not observed on TBB in 36% of 25 CEP patients with BAL eosinophilia (1,20). Again, BAL eosinophilia values above 5% can be considered significant among the diagnostic criteria in the literature. However, there are also patients without BAL eosinophilia in the literature. As reported by Taniguchi et al. and others, cases of CEP without BAL eosinophilia have been documented in the literature (1,24). It is also possible that these patients may have used steroids before BAL due to asthma attack-like symptoms. Therefore, the diagnosis of patients should not rely solely on clinical symptoms, radiological findings, peripheral eosinophilia, or bronchoalveolar lavage results. It is essential to consider their medical history, asthma status, all relevant parameters, and carefully exclude other potential diagnoses before reaching a conclusion. The patient with p ANCA positivity was evaluated in detail in terms of EGPA both at the time of diagnosis and during follow-up, consulted to the rheumatology clinic, no evidence of vasculitis was found and it was observed that the patient did not meet the diagnostic criteria for EGPA (25,26).

Inhaled corticosteroids are not sufficient when a patient experiences CEP symptoms. All of our patients reported using ICS. CEP is traditionally treated with systemic corticosteroid therapy. However, in most

cases, especially in asthmatic patients, relapses can be seen when systemic corticosteroid use is reduced by more than 50% (14-16). In a study conducted in 133 CEP cases, two or more recurrences were found in 28.6% (27) of the patients.

The glucocorticoid-sparing effect of omalizumab has been reported in patients with severe allergic and non-allergic asthma (28). These data are also valid for patients with CEP on a case-by-case basis. When the literature is examined, Kaya et al. first demonstrated the efficacy of Omalizumab in CEP cases on a case basis (13). Later, Eduardo et al. presented a positive response of Omalizumab in a steroid-refractory CEP case (3,13,29). Our study represents the largest series in this regard among CEP patients. The presence of concomitant asthma diagnoses may introduce complexity when assessing the efficacy of omalizumab. However, our primary focus was to evaluate the reduction of relapses in CEP and the potential for decreasing or discontinuing steroid therapy. It is the number of simultaneous peripheral eosinophilia. However, our primary focus was to reduce relapses in CEP and to avoid concomitant increase in peripheral eosinophilia while considering the possibility of tapering or discontinuation of steroid therapy. Symptom control, pulmonary function follow-up and improvement in asthma attacks were secondary outcomes.

Omalizumab functions by reducing the expression of the Fc epsilon RI alpha receptor on basophils. In addition, regardless of IgE levels, tryptase reduces Th₂ cytokines (IL-4 and IL-13) and chemokines (IL-8 and RANTES). Similarly, it decreases cytokine expression in dendritic cells (IL-5, IL-10, and IL-13) and interferes with their ability to activate CD4. It also decreases eosinophil counts, monocytes, FENO, and endothelin-1 concentrations in sputum, expiratory air, and lung tissue. In addition, stimulation of IgE increases the deposition of collagen I, III, and IV and fibronectin along the Erk1/2 MAPK pathway, thereby enhancing airway remodeling (30-32). Noga et al. found that the eosinophil apoptosis marker Annexin V was significantly higher after anti-IgE treatment (33). They attributed the efficacy of omalizumab to the increase in eosinophil apoptosis and the decrease in GM-CSF (33).

There is also evidence indicating that blocking IgE with omalizumab has an inhibitory effect on eosinophils. Djukanovic et al. reported that omalizumab significantly reduced the sputum and

bronchial submucosal eosinophil counts in patients with allergic asthma (34). Furthermore, in a study conducted by Massarani M et al., it was demonstrated that treatment with omalizumab led to a significant reduction in peripheral blood eosinophilia compared to placebo (35).

With all these findings considered, it is evident that omalizumab exerts effects beyond its role as an IgE immunomodulator, and its impact on eosinophilic inflammation is of significant importance. The fact that the eosinophil level remained within certain ranges and did not increase during the reduction and discontinuation of steroid therapy in our patients further confirms these effects. Positive responses to treatment have been reported in conditions such as EGPA, as in the CEP form of eosinophilic inflammation (16,36). While we concur with this perspective, there is indeed a requirement for comprehensive studies involving a large number of cases to elucidate the mechanism of action thoroughly. In other biological agents, of course, CEP is also used on a case-by-case basis. There are case series about the anti-IL5/IL5R monoclonal agents mepolizumab and benralizumab that are also effective in CEP (37-39). Results from ten cases of CEP were presented in a recent multicenter study involving mepolizumab. It has been reported that steroid use was discontinued after three months in nine patients and continued at 2.5 mg in one patient. Case reports have been documented regarding dupilumab-related side effects and exacerbation of symptoms associated with CEP during its use (40). Randomized controlled trials evaluating the efficacy of biological therapies in treating CEP are necessary. The long-standing use and relatively lower cost of omalizumab compared to other biological agents may contribute to its preference in clinical practice.

In our study, a substantial proportion of patients responded to treatment. Other case series were analyzed to determine the drug dose of the patients. As in EGPA, non-atopic asthma, and other cases, anti-IgE-based biological therapy was adjusted according to IgE level and weight. It is a real-life study with a mean follow-up period of 22 months. It differs from other studies in terms of long-term follow-up. One of the limitations of the study is its open-label nature. Although the placebo effect is undeniable, objective improvement was observed in patients’ symptoms, respiratory function, relapse rates, and hospitalization rates. At the same time, the

small sample size, the decrease in the number of patients followed up in the second and third years, and the absence of a control group are other limitations, a circumstance possibly compounded by the rarity of the disease.

## CONCLUSION

Omalizumab may represent an effective treatment for CEP and may be considered as a steroid-sparing agent.

**Ethical Committee Approval:** This study was approved by the Ankara Keçiören Training and Research Hospital Clinical Research Ethics Committee (Decision no: 2012-KAEK-15/1744, Date: 12.09.2018).

## CONFLICT of INTEREST

The authors declare no potential conflicts of interest with respect to research, authorship and/or publication of this article.

## AUTHORSHIP CONTRIBUTIONS

Concept/Design: FÖE, KA, BBA, İKK Analysis/Interpretation: BBA, ÖA, HA, İKK, GKB Data acqusition: BBA, ÖA, HA, İKK, GKB Writing: BBA, KA, FÖE

Clinical Revision: KA, FÖE, İKK, GKB Final Approval: All of authors

## REFERENCES

1. *
  Suzuki Y, Suda T. Eosinophilic pneumonia: A review of the previous literature, causes, diagnosis, and management. Allergol Int 2019; 68(4): 413-9. https://doi.org/10.1016/j. alit.2019.05.006*
2. *
  Sveinsson OA, Isaksson HJ, Gudmundsson G. Chronic eosinophilic pneumonia in Iceland: Clinical features, epi- demiology and review. Laeknabladid 2007; 93: 111-6.
  *
3. *
  Shin Y, Jin HY, Yoo H, Hwang E, Nam Y, Ye Y, et al. Successful treatment of chronic eosinophilic pneumonia with Anti-IgE therapy. J Korean Med Sci 2012; 27: 1261-4. https://doi.org/10.3346/jkms.2012.27.10.1261*
4. *
  Djukanovic R, Wilson SJ, Kraft M, Jarjour NN, Steel M, Chung KF, et al. Effects of treatment with anti-immuno- globulin E antibody omalizumab on airway inflammation in allergic asthma. Am J Respir Crit Care Med 2004; 170: 583-93. https://doi.org/10.1164/rccm.200312-1651OC*
5. *
  Foroughi S, Foster B, Kim N, Bernardino LB, Scott LM, Hamilton RG, et al. Anti-IgE treatment of eosinophil-asso- ciated gastrointestinal disorders. J Allergy Clin Immunol 2007; 120: 594-601. https://doi.org/10.1016/j. jaci.2007.06.015*
6. *
  Nam YH, Kim JH, Jin HJ, Hwang EK, Shin YS, Ye YM, et al. Effects of omalizumab treatment in patients with refracto- ry chronic urticaria. Allergy Asthma Immunol Res 2012; 4(6): 357-61. https://doi.org/10.4168/aair.2012.4.6.357*
7. *
  Plewako H, Arvidsson M, Petruson K, Oancea I, Holmberg K, Adelroth E, et al. The effect of omalizumab on nasal allergic inflammation. J Allergy Clin Immunol 2002; 110: 68-71. https://doi.org/10.1067/mai.2002.125488*
8. *
  Yorgancıoğlu A, Öner Erkekol F, Mungan D, Erdinç M, Gemicioğlu B, Özşeker ZF, et al. Long-term omalizumab treatment: A multicenter real-life, 5-year trial. Int Arch Allergy Immunol 2018; 176: 225-33. https://doi. org/10.1159/000488349*
9. *
  El-Qutob D. Off-label uses of Omalizumab. Clin Rev Allergy Immunol 2016; 50(1): 84-96.
  *

*https://doi.org/10.1007/s12016-015-8490-y*

10. *
  Aksu K, Kurt E. Aspirin tolerance following omalizumab therapy in a patient with aspirin-exacerbated respiratory disease. Allergol Immunopathol 2013; 41(3): 208-10. https://doi.org/10.1016/j.aller.2012.02.005*
11. *
  Aydın O, Sozener ZC, Soyyiğit S, Kendirlinan R, Gençtürk Z, Mısırlıgil Z. Omalizumab in the treatment of allergic bronchopulmonary aspergillosis: One center’s experience with 14 cases. Allergy Asthma Proc 2015; 36(6): 493-500. https://doi.org/10.2500/aap.2015.36.3909*
12. *
  Çelebi Sözener Z, Aydın Ö, Mısırlıgil Z, Mungan D, Demirel YS, Çelik G, et al. Omalizumab in nonallergic asthma: A report of 13 cases. J Asthma 55: 7; 756-63. https://doi.org/10.1080/02770903.2017.1362427*
13. *
  Kaya H, Gumus S, Ucar E, Aydogan M, Musabak U, Tozkoparan E, et al. Omalizumab as a steroid-sparing agent in chronic eosinophilic pneumonia. Chest J 2012; 142(2): 513-6. https://doi.org/10.1378/chest.11-1881*
14. *
  Garcia G, Magnan A, Chiron R, Contin-Bordes C, Berger P, Taillé C, et al. A proof-of concept, randomized, controlled trial of omalizumab in patients with severe, difficult-to-con- trol,nonatopic asthma. Chest 2013; 144: 411-9. https:// doi.org/10.1378/chest.12-1961*
15. *
  Pillai P, Chan YC, Wu SY, Ohm-Laursen L, Thomas C, Durham SR, et al. Omalizumab reduces bronchial muco- sal IgE and improves lung function in non-atopic asthma. Eur Respir J 2016; 48: 1593-601. https://doi. org/10.1183/13993003.01501-2015*
16. *
  Bianca O, Elisa T, Marco C, Claudio L. Biologics for the treatment of allergic conditions: Eosinophil disorders. Immunol Allergy Clin N Am 2020; 40: 649-65. https://doi. org/10.1016/j.iac.2020.07.001*
17. *
  Pavord ID, Bel EH, Bourdin A, Chan R, Han JK, Keene ON, et al. From DREAM to REALITI-A and beyond: Mepolizumab for the treatment of eosinophil-driven diseases. Allergy 2022; 77(3): 778-9. https://doi.org/10.1111/all.15056*
18. *
  Pitlick MM, Li JT, Pongdee T. Current and emerging biolog- ic therapies targeting eosinophilic disorders World Allergy Organ J 2022; 15: 1006-76. https://doi.org/10.1016/j. waojou.2022.100676*
19. *
  Cottin V. Eosinophilic lung diseases. Clin Chest Med 2016; 37(3): 535-56. https://doi.org/10.1016/j. ccm.2016.04.015*
20. *
  Matsuse H, Shimoda T, Fukushima C, Matsvo N, Sakai H, Takao A, et al. Diagnostic problems in chronic eosinophil- ic pneumonia. J Int Med Res 1997; 25: 196-201. https:// doi.org/10.1177/030006059702500404*
21. *
  James A, Michael W. Eosinophilic pneumonias. J Allergy Clin Immunol Pract 2018; 6(5): 1455-61.
  *
22. *
  Carrington CB, Addington WW, Goff AM, Madoff JI, Marks A, Schwaber JR, et al. Chronic eosinophilic pneumonia. N Engl J Med 1969; 280: 787-98. https://doi.org/10.1056/ NEJM196904102801501*
23. *
  Jederlinic PJ, Sicilian L, Gaensler EA. Chronic eosinophilic pneumonia. A report of 19 cases and a review of the liter- ature. Medicine (Baltimore) 1988; 67: 154-62. https:// doi.org/10.1097/00005792-198805000-00002*
24. *
  Taniguchi H, Kadota J, Abe K, Matsubara Y, Kaseda M, Kawamoto S, et al. Eosinophilic pneumonia without eosin- ophilia in BALF or peripheral blood and diagnosed by open lung biopsy. Nihon Kokyuki Gakkai Zasshi 1999; 37: 796-801 (in Japanese).
  *
25. *
  Gümüşburun R, Mungan D, Kayacan O, Perçinel S, Bavbek S. Eosinophilic granulomatosis with polyangiitis and chronic eosinophilic pneumonia: Similarities and dif- ferences. Asthma Allergy Immunol 2018; 16: 112-7.
  *
26. *
  Argüder E, Çelik GE, Bavbek S, Göksel ÖK, Mısırlıgil Z. A case with eosinophilic lung diseases: chronic eosinophilic pneumonia or Churg-Strauss syndrome? Asthma Allergy Immunol 2009; 7: 74-8.
  *
27. *
  Naughton M, Fahy J, FitzGerald MX. Chronic eosinophilic pneumonia. A long-term follow-up of 12 patients. Chest J 1993; 103: 162-5. https://doi.org/10.1378/ chest.103.1.162*
28. *
  Stokes JR, Casal TB. The use of Anti-IgE therapy beyond allergic asthma. J Allergy Clin Immunol Pract 2015; 3: 162-6. https://doi.org/10.1016/j.jaip.2014.10.010*
29. *
  Eduardo L, Ana A, José Luis IA. Response to omalizumab in a patient with chronic eosinophilic pneumonia and poor response to corticosteroids. Arch Bronconeumol 2018; 54(7): 390-401. https://doi.org/10.1016/j. arbr.2018.05.004*
30. *
  Takaku Y, Soma T, Nishihara F, Nakagome K, Kobayashi T, Hagiwara K, et al. Omalizumab attenuates airway inflam- mation and interleukin-5 production by mononuclear cells in patients with severe allergic asthma. Int Arch Allergy Immunol 2013; 161 (Suppl 2): 107-17. https:// doi.org/10.1159/000350852*
31. *
  Zietkowski Z, Skiepko R, Tomasiak-Lozowska MM, Bodzenta-Lukaszyk A. Anti-IgE therapy with omalizumab decreases endothelin-1 in exhaled breath condensate of patients with severe persistent allergic asthma. Respiration 2010; 80: 534-42. https://doi.org/10.1159/000317137*
32. *
  Roth M, Zhong J, Zumkeller C, Song CT, Goulet S, Tamm
  *

*
M. The role of IgE-receptors in IgE-dependent airway smooth muscle cell remodelling. PLOS ONE 2013; 8: 560-
*

*
5. https://doi.org/10.1371/journal.pone.0056015*

33. *
  Noga O, Hanf G, Brachmann I, Klucken AC, Kleine-Tebbe J, Rosseau S, et al. Effect of omalizumab treatment on peripheral eosinophil and T-lymphocyte function in patients with allergic asthma. J Allergy Clin Immunol 2006; 117: 1493-9. https://doi.org/10.1016/j. jaci.2006.02.028*
34. *
  Skiepko R, Ziętkowski Z. Changes in blood eosinophilia during omalizumab therapy as a predictor of asthma exac- erbation. Postepy Dermatol Alergol 2014; 31(5): 305-9. https://doi.org/10.5114/pdia.2014.40973*
35. *
  Massanari M, Holgate ST, Busse WW, Jimenez P, Kianifard F, Zeldin R. Effect of omalizumab on peripheral blood eosinophilia in allergic asthma. Respir Med 2010; 104(2): 188-96. https://doi.org/10.1016/j.rmed.2009.09.011*
36. *
  Celebi Sozener Z, Gorgulu B, Mungan D, Sin AB, Misirligil Z, Aydin Ö, et al. Omalizumab in the treatment of eosino- philic granulomatosis with polyangiitis (EGPA): Single- center experience in 18 cases. World Allergy Organ J 2018; 11(1): 39. https://doi.org/10.1186/s40413-018-0217-0*
37. *
  Brenard E, Pilette C, Dahlqvist C, Colinet B, Schleich F, Roufosse F, et al. Real-life study of mepolizumab in idio- pathic chronic eosinophilic pneumonia. Lung 2020; 198(2): 355-60. https://doi.org/10.1007/s00408-020- 00336-3*
38. *
  Isomoto K, Baba T, Sekine A, Aiko N, Ogura T. Promising effects of benralizumab on chronic eosinophilic pneumo- nia. Intern Med 2020; 59(9): 1195-8. https://doi. org/10.2169/internalmedicine.3606-19*
39. *
  Shimizu Y, Kurosawa M, Sutoh Y, Sutoh E. Long-term treat- ment with anti-interleukin 5 antibodies in a patient with chronic eosinophilic pneumonia. J Investig Allergol Clin Immunol 2020; 30(2): 154-5. https://doi.org/10.18176/ jiaci.0468*
40. *
  Menzella F, Montanari G, Patricelli G, Cavazza A, Galeone C, Ruggiero P. A case of chronic eosinophilic pneumonia in a patient treated with dupilumab. Ther Clin Risk Manag 2019; 15: 869-75. https://doi.org/10.2147/TCRM. S207402*

